# Nitrogen-Centered Radicals Derived from Azidonucleosides

**DOI:** 10.3390/molecules29102310

**Published:** 2024-05-14

**Authors:** Yahaira Reyes, Amitava Adhikary, Stanislaw F. Wnuk

**Affiliations:** 1 Department of Chemistry and Biochemistry, Florida International University, Miami, FL 33199, USA; yreye008@fiu.edu; 2 Department of Chemistry, Oakland University, Rochester, MI 48309, USA; adhikary@oakland.edu

**Keywords:** aminyl radicals, azides, iminyl radicals, nitrogen-centered radicals, nucleosides, purines, pyrimidines, radiation, radiosensitizers, ribonucleotide reductases

## Abstract

Azido-modified nucleosides have been extensively explored as substrates for click chemistry and the metabolic labeling of DNA and RNA. These compounds are also of interest as precursors for further synthetic elaboration and as therapeutic agents. This review discusses the chemistry of azidonucleosides related to the generation of nitrogen-centered radicals (NCRs) from the azido groups that are selectively inserted into the nucleoside frame along with the subsequent chemistry and biological implications of NCRs. For instance, the critical role of the sulfinylimine radical generated during inhibition of ribonucleotide reductases by 2′-azido-2′-deoxy pyrimidine nucleotides as well as the NCRs generated from azidonucleosides by radiation-produced (prehydrated and aqueous) electrons are discussed. Regio and stereoselectivity of incorporation of an azido group (“radical arm”) into the frame of nucleoside and selective generation of NCRs under reductive conditions, which often produce the same radical species that are observed upon ionization events due to radiation and/or other oxidative conditions that are emphasized. NCRs generated from nucleoside-modified precursors other than azidonucleosides are also discussed but only with the direct relation to the same/similar NCRs derived from azidonucleosides.

## 1. Introduction

Nitrogen-centered radicals (NCRs) play an important role in chemical biology and cellular signaling [1,2,3] as well as in organic synthesis, as they allow access to new synthetic pathways in nonconventional ways [4,5,6,7,8]. NCRs are categorized into four main types of radicals: σ-iminyl (R=N·), π-aminyl (R^1^-N(·)-R^2^), π-amidyl (R^1^-CO-N(·)-R^2^), and π-aminium (R^1^-N(·)H-R^2^)^+^. They are generated via homolytic cleavage, reductive/oxidative conditions, and proton-coupled electron transfer (PCET) methods [4,5,7,8]. Precursors to NCRs include *N*-halogenated amines [9,10], aryloxyamides [11], sulfonylamides [12], or *O*-aroyloximes [13], among others [4,8]. NCRs generated from azides (R-N_3_) are important due to the synthetic ease of the incorporation of azido groups into the frame of complex molecules, including natural products and their versatile application to the subsequent functionalization reactions [14,15].

Azido-modified nucleosides have been of interest for over six decades, and the finding that 3′-azido-3′-deoxythymidine (AZT) is a therapeutic agent [16,17] for the treatment of acquired immunodeficiency syndrome (AIDS) has sparked attention to their chemistry. The synthesis of azidonucleosides, their reactions, and biological activities have been subject of comprehensive reviews [18,19,20]. Azidonucleosides have been explored as substrates for the (a) synthesis of amino nucleosides [21], (b) click chemistry [20], (c) bioconjugation and ligation [19], (d) the metabolic labeling of DNA and RNA and for live cell fluorescence imaging [22], and (e) radical biology including enzyme inhibitions [23,24], among others [18,25].

In the last twenty years, azido-modified nucleosides, nucleotides, and oligonucleotides have been extensively explored as substrates for click chemistry [26,27,28,29]. In general, the application of the azidonucleosides and oligonucleotides in click chemistry is more demanding than their alkyne counterparts due to the challenging chemical [30,31,32,33,34] and enzymatic [35] synthesis and their lack of compatibility with the solid-phase synthesis of DNA fragments [36,37]. Also, the triazole click products are often used for the fluorescence imaging of cancer cells [38,39,40] and for cross-linking cellular nucleic acids [41]. The azide group can often cyclize with nitrogen atoms present in the pyrimidine or purine ring to form tetrazole tautomers [42,43]. Moreover, 5-azidouracil and 8-azidoadenine have also been utilized as photoaffinity probes. Upon UV-irradiation, these aryl azides produce highly reactive nitrenes that can react with most amino acid residues, thereby resulting in nucleic acid–protein photo-crosslinking in yields above 50% [25].

Recently, 2′-deoxy-2′-β-fluoro-4′-azidocytidine (Azvudine), a clinical candidate originally developed for HIV treatment, entered clinical trials in China for evaluating its efficacy and safety and showed promise for treating coronavirus disease (COVID-19) [44,45]. Moreover, 4′-azidocytidine is a potent inhibitor of HCV (R1479) [46]. Azido-substituted nucleosides have been developed for use as prodrugs [42,47], as adenosine receptor antagonists [48], for determining protein–DNA/RNA interactions [34,49], or as anticancer [47] and anti-viral agents [50,51].

Furthermore, 3′-azido-3′-deoxythymidine (AZT) has been employed as a radiation sensitizer in the radiotherapy of tumors for HIV-positive patients [52]. AZT demonstrated significant radiosensitization in irradiated human colon cancer, larynx squamous carcinoma, and malignant glioma cells [53,54,55]. The aim of this account is to discuss the application of azidonucleosides in the selective and site-specific generation of NCRs and their biological implications. Pulse radiolysis, photolysis, and electrochemical investigations as well as enzymatic and biomimetic model studies in combination with electron paramagnetic resonance (EPR) and density functional theory investigations were employed to elucidate the formation of NCRs in azidonucleosides and their subsequent reactions. Thus, the focus of this review is not on the synthesis of azidonucleosides but rather on the importance of the chemistries of NCRs generated from the azido group. NCRs generated from non-azidonucleoside-modified precursors, by ionization, or by the one-electron oxidation of the parent nucleobase are discussed only in relation (comparison) to the azido-derived radical chemistry.

### 1.1. 2′-Azido-2′-Deoxy Pyrimidine Nucleosides and Nucleotides: Inhibition of Ribonucleotide Reductases and Importance of Nitrogen-Centered Radical Chemistry

**Figure 1:**
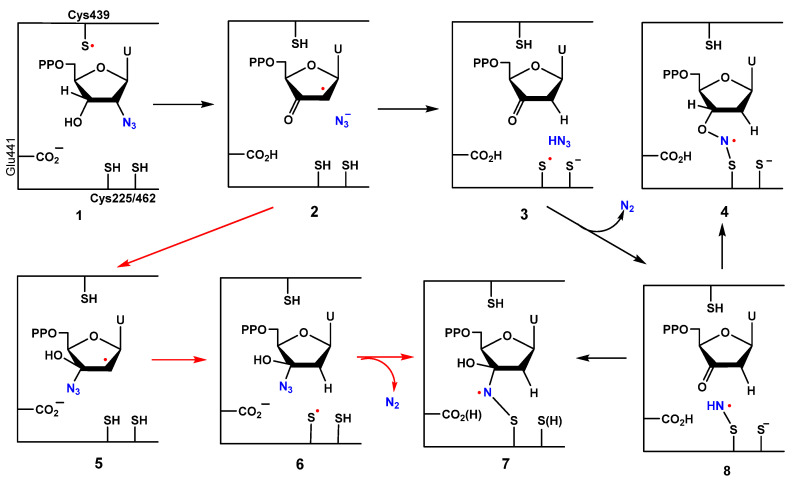
Proposed structures for the nitrogen-centered radicals (NCRs) and pathways for their generation during the inactivation of RDPR by N_3_UDP [57,58,59,65].

2′-Azido-2′-deoxynucleoside 5′-diphosphates (e.g., **1**, N_3_UDP) are potent inactivators of ribonucleotide reductases (RDPR). Sjöberg et al. found that the inactivation of RDPR by 2′-azido-2′-deoxynucleotides was accompanied by the appearance of new EPR signals for a nitrogen-centered radical (anisotropic triplet with a second hyperfine interaction) and the concomitant decay of peaks for the tyrosyl radical [56], which was the first direct evidence for free radical chemistry with RDPR. The structure of this elusive nitrogen radical was studied extensively and shown to be derived from the azide moiety [57,58,59]. A proposed mechanism postulated azide loss (as an anion) from the initial C3′ radical intermediate to give the ketyl radical **2** and subsequent reduction by proton-coupled electron transfer to generate the 2′-deoxy-3′-ketonucleotide **3** (Figure 1). This process leaves a thiyl radical in the active site. The reaction of hydrazoic acid with the thiyl radical generates stoichiometric N_2_ and a sulfinylimine radical **8**. The protonated azide (p*K*_a_ of 4.6) was hypothesized to be essential for that mechanism [57,59]. Conversion (**2 → 3**) is analogous to the proposed mechanism for the reduction of natural nucleotides that proceeds by the generation of the same 3′-keto-2′-deoxynucleotide intermediate, which makes the investigation of the inhibition of RDPR by N_3_UDP even more significant [23,60]. The initial NCR **8** reacts further with the oxygen or carbon atoms of a carbonyl group of the 3′-keto-2′-deoxynucleotide to generate radicals **4** or **7**, respectively [58].

The inactivation of the RDPR with 3′[^17^O]-N_3_UDP [61] **1** was consistent with the formation of the radical, R-S-N·-C(3′)-OH **7**, and provided the first evidence for the trapping of a 3′-ketonucleotide in the reduction process by a nitrogen-centered radical **8** [59]. Chemical requirements also favor the formation of **7** (over **4**), and there is precedent in the literature for the analogous addition of aminyl radicals to carbonyl [62] and imino groups [63]. Moreover, the inactivation of the adenosylcobalamine-dependent RTPR with 2′-*arabino*-2′-azido-2′-deoxyadenosine-5′-triphosphate was accompanied by the detection of a paramagnetic species by EPR spectroscopy. In a tentatively proposed mechanism, perhaps due to an altered sugar pucker and the steric constraints imposed by the azido moiety on the β-face of the nucleoside, the C2′-azide was acting here as a radical trap for the initially formed protein thiyl radical to generate a sulfinylimine NCR [64].

The theoretical modeling study of the inactivation of RDPR by N_3_UDP by Pereira and coworkers generated an alternative mechanistic proposal (depict by red arrow path, Figure 1) [65]. In that hypothesis, the released azide anion (N_3_^−^) was proposed to add to the 2′-ketyl radical **2** first with the concomitant protonation of the ketone oxygen by E441. The resulting radical **5** is then reduced at the 2′ position by Cys225 to generate the Cys225 thiyl radical **6**. A subsequent attack of the thiyl radical on an alkyl azide (instead of HN_3_ or N_3_^−^) would lead to the same nitrogen-centered radical **7** that was detected experimentally [59]. The addition of azide to ketones has chemical precedence [66], but there are no model systems where azide adds to ketyl radicals. The reduction of alkyl azides with tin [62,67], alkoxy [68], and silyl (in the presence of thiols) [69,70] radicals have been reported [15].

### 1.2. Biomimetic Studies

**Figure 2:**
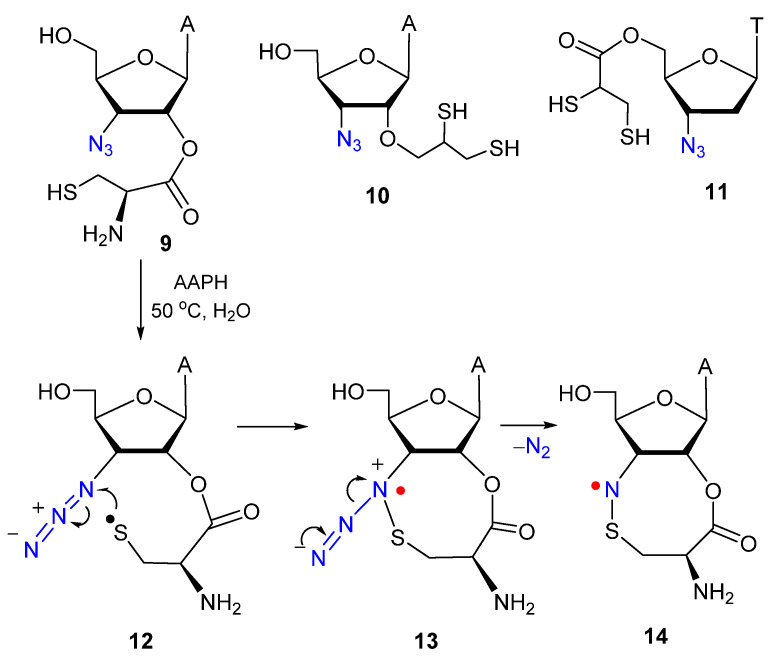
Model azidonucleosides with the cysteinate or vicinal dithiol unit attached to C2′ or C5′ to study the assumed ring closure reactions between the thiyl radical and the azido group. Plausible intramolecular interactions of the thiyl radical with an azido group at C3′ for adenosine-derived substrate **9** [71].

Thao and coworkers designed model 3′-azido-3′-deoxynucleosides with thiol or vicinal dithiol substituents at C2′ or C5′ to study reactions postulated to occur during the inhibition of ribonucleotide reductases (RNRs) by N_3_UDP (**9**–**11**, Figure 2) [71]. It was presumed that the intramolecular addition of the thiyl radical in **12** (generated from **9**) to the azido group via an eight-membered TS would produce the transient triazenyl radical **13**. A loss of N_2_ would generate nitrogen-centered radical **14** (similar to sulfinylimine radical of type **7**; vide supra), and an abstraction of a hydrogen atom by **14** should give a cyclic sulfenamide, which might undergo ring opening to give 3′-amino-3′-deoxy products. Density functional theory calculations predicted that intramolecular reactions between the generated thiyl radicals and azido group on the model compounds **9**–**11** will be exothermic by 33.6–41.2 kcal/mol with low energy barriers of 10.4–13.5 kcal/mol [71].

The heating of 3′-azido-3′-deoxy-5-*O*-(2,3-dimercaptopropanoyl)thymidine **11** in H_2_O with 2′-azobis-(2-methyl-2-propionamidine) dihydrochloride (AAPH) as an initiator for the production of thiyl radicals gave 3′-amino-3′-deoxythymidine, whereas the analogous treatment of 3′-azido-3′-deoxythymidine (AZT, a control substrate lacking a thiol substituent) resulted in the isolation of major quantities of unchanged AZT. Moreover, the γ-radiolysis of N_2_O-saturated aqueous solutions of AZT and cysteine produced 3′-amino-3′-deoxythymidine and thymine. The DFT-calculated predictions and results with radical-initiated intramolecular azido substituent reduction with model compounds, such as **9** and **11**, bearing azido and thiol substituents might be in harmony with the enzymatic positioning of azido-containing substrates in close proximity with thiol functionalities that exist in the active sites of RNRs [71].

**Figure 3:**
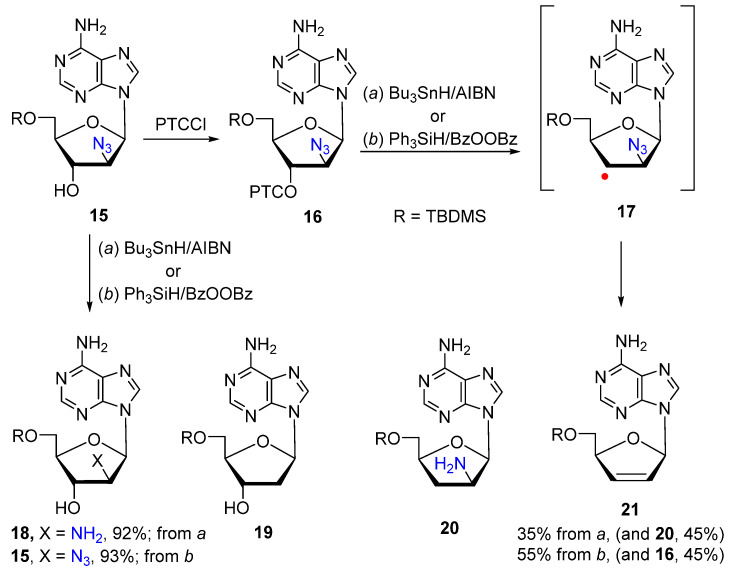
Differential interaction of azidonucleosides with tributylstannane and triphenylsilane. Reduction versus elimination of azido group from C2′ position [73].

Robins and coworkers explored further biomimetic reactions to occur during the mechanism-based inhibition of RDPR by N_3_UDP with model 2′-substituted nucleoside derivatives [72], which can cause the elimination of radical or ionic species from C2′ upon the generation of a radical at C3′, including 2′-azido-substituted uridine and adenosine models (e.g., **15** [21]) [73]. Azide **15** was converted to 3′-*O*-phenoxythiocarbonyl (PTC) derivative **16**. Compounds **15** and **16** were subjected to parallel treatment with tributylstannane/AIBN/toluene/Δ (Method *a*) and also with triphenylsilane/dibenzoyl peroxide/toluene/Δ (Method *b*; Figure 3). The known stannyl “radical-mediated” reduction [74] of azide **15** to amine **18** occurred with tributylstannane/AIBN. Interestingly, a reduction of azide **15** to amine **18** was not observed with triphenylsilane/dibenzoyl peroxide. It is also noteworthy that although the radical-mediated cleavage of the carbon-nitrogen bond with azides is unknown, the treatment of the *ribo* epimer of **15** with excess triphenylsilane and a prolonged reaction time caused some dehydrogenolytic deazidation to give small quantities of the 2′-deoxy derivative **19** in the absence of amine formation [73].

The treatment of 3′-thionocarbonate **16** with Bu_3_SnH resulted in radical-mediated elimination upon the generation of a radical at C3′ **17** to give 2′,3′-didehydro-2′,3′-dideoxy nucleoside **21** in moderate yields, but competing reduction of the azido group and hydrogenolysis of the thionocarbonate group also produced byproduct **20**. Elimination product **21** was formed almost exclusively upon the treatment of **16** with Ph_3_SiH but at a lower conversion rate and with the recovery of starting material **16** (Figure 3) [73].

## 2. Nitrogen-Centered Radicals Generated from Azidonucleosides by Radiation-Produced Electrons

**Scheme 1:**
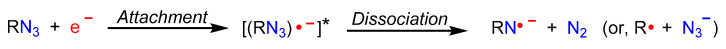
Schematic representation of the steps involved in the dissociate electron attachment process involving the neutral azide molecule, RN_3_. The first step, attachment, leads to the formation of a transient negative ion (TNI), [(RN_3_)·^−^]*. This is followed by dissociation [78].

Azide anion (N_3_^−^) has a low reactivity with radiation-produced electrons (e.g., with aqueous electrons, i.e., with fully solvated electrons when water is the solvent) [75]; however, the aqueous electrons react with azidonucleosides with an almost diffusion-controlled rate (~10^10^ M^−1^s^−1^), forming NCRs [76]. The plausible involvement of dissociative electron attachment pathway in the formation of NCRs from azidonucleosides could be inferred from the dissociative electron attachment (DEA) spectra of phenyl azide (Scheme 1). Studies establish that the major fragmentation pathway involves N_2_ loss [77].

### 2.1. From Azido-Modified Nucleoside Sugars

**Figure 4:**
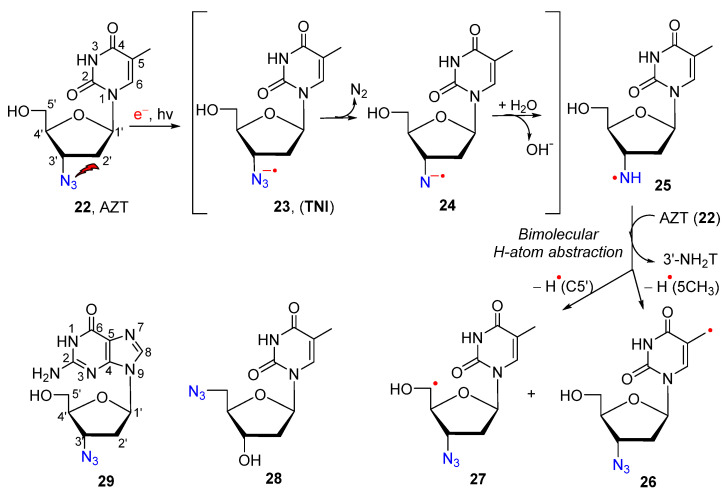
The formation and reactivity of the aminyl radical generated upon the addition of radiation-produced electrons to AZT [79].

Sevilla and Adhikary developed a method for the generation of aminyl radicals on dissociative electron attachment (DEA) to azidonucleosides, which allows for detailed EPR spectroscopic studies of aminyl radicals and their subsequent chemistry [79]. EPR spectral studies and density functional theoretical calculations showed that the predominant site of electron capture in AZT (**22**) is at the azide group (ca. 80%) and not at the thymine moiety (ca. 20%), which is the most electron-affinic DNA-base [79]. In this work, the radiation-produced prehydrated electrons led to the site-specific formation of a localized π-aminyl radical (RNH·) **25** [in homogeneous glassy 7.5 M aqueous (D_2_O or H_2_O) LiCl solutions; Figure 4]. It was demonstrated that the neutral azide can capture an electron due to its high electron affinity and form a transient negative ion (TNI), an unstable azide anion radical RN_3_·^−^, **23** (see also Scheme 1) when irradiated. RN_3_·^−^ can then facilely lose N_2_, leaving a nitrene anion radical (RN·^−^, **24**; neither were detected by EPR even at 77 K) that upon protonation from the surrounding solvent becomes a neutral π-aminyl radical (RND·/RNH·, **25**), which was detected by EPR. Upon annealing to higher temperatures (ca 160–170 K), RNH· **25** undergoes bimolecular H-atom abstraction from the C5-methyl group of **22**, generating the allyl radical **26** or from the sugar moiety yielding C5′· (**27**) [79].

**Figure 5:**
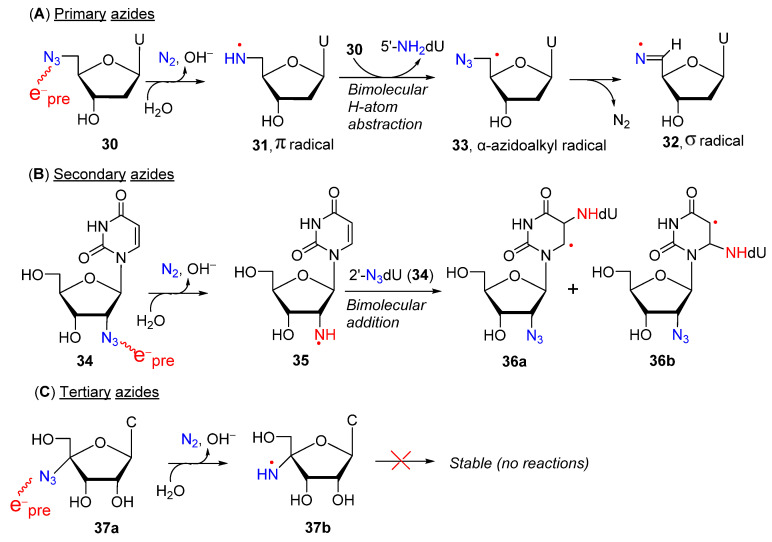
Types of reactions undergone by π-RNH· attached to a primary (path **A**), secondary (path **B**), or tertiary (path **C**) alkyl carbon in the ribose sugar of pyrimidine nucleosides.

A radiation-produced electron addition to 5′-azido-5′-deoxythymidine **28** forms C5′-NH·, which undergoes predominant (ca. 80%) bimolecular H-atom abstraction from a proximate C5-methyl group generating the allyl radical (of type **26**) and ca. 20% of the σ-type iminyl radical in the sugar moiety (similar to **32**; see Figure 5 and relevant discussion) [79,80]. Interestingly, electron attachment to 3′-azido-2′,3′-dideoxyguanosine **29** results in the one-electron oxidation of the guanine base via an intramolecular electron-coupled proton transfer pathway to give the G(N1-H)· radical [79,81] but not the elusive guanyl radical G(N_2_-H)· [82]. The latter is formed via the deprotonation of a guanyl cation radical from the exocyclic amine of the guanine base in DNA but not in monomers [79,83,84].

Mudgal et al. reported that the site of azido substitution in the sugar moiety of azidopyrimidine nucleosides influences the reactivity of aminyl radicals formed by dissociative electron attachment (Figure 5) [80]. Employing a ^15^N-labeled azido group and deuterations at specific sites in the sugar and base, it was shown that they initially form aminyl radicals RNH· upon annealing samples from 77 K to 170 K: (*i*) at a primary carbon site (e.g., **31**; generated from 5′-azido-2′,5′-dideoxyuridine, **30**), it is converted to a σ-type iminyl radical (R=N·) **32** via a concentration-dependent bimolecular H-atom abstraction reaction between **30** and **31** and subsequent nitrogen loss from the intermediary α-azidoalkyl radical **33** (path A); (*ii*) at a secondary carbon site RNH· **35**, it is generated from 2′-azido-2′-deoxyuridine (**34**, 2′-N_3_dU) and underwent bimolecular electrophilic addition to the C5=C6 double bond of a proximate pyrimidine base to give C6 **36a** and C5 **36b** radicals (path B); and (*iii*) at tertiary alkyl carbon (e.g., in 4′-azidocytidine **37a**), RNH· **37b** is quite stable and undergoes little to no reaction (path C). These results show the influence of the stereochemical and electronic environment on RNH· reactivity and potentially should allow for the selection of azidonucleosides, which would be the most effective in augmenting cellular radiation damage [80].

**Figure 6:**
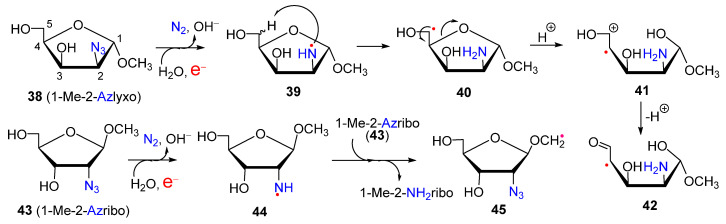
The formation of the ring-opened C4· **42** via the ring opening of a C5· intermediate in 1-Me-2-Azlyxo (**38**) and thermodynamically stable -OCH_2_· **45** with an intact ribofuranose ring in 1-Me-2-Azribo (**43**).

To further test the mechanism for sugar radical formation from the π-aminyl radicals without the nucleobase interaction, Mudgal et al. investigated the formation and reactivity of the π-aminyl radicals from azidopentafuranoses [e.g., methyl 2-azido-2-deoxy-α-D-lyxofuranoside, **38** (1-Me-2-Azlyxo), and its β-D-ribo isomer **43** (1-Me-2-Azribo)] [85]. Prehydrated electron attachment to **38** and its ^15^N- and ^2^H-labeled derivatives showed unequivocal (concentration independent) intramolecular H-atom abstraction from the C5 by RNH· in **39** via a favorable six-membered transition state ([1,5]-hydrogen shift) to produce the primary α-hydroxy C5· **40**. Subsequent ring opening (**40** → **41**) and unimolecular conversion produced a secondary C4· **42** under the reductive environment of DEA (Figure 6) [85]. However, for **43**, EPR studies established thermally-activated (concentration dependent) intermolecular H-atom abstraction by RNH· **44** from the methyl group at the C1 of **43** to generate the carbon-centered radical **45**.

**Figure 7:**
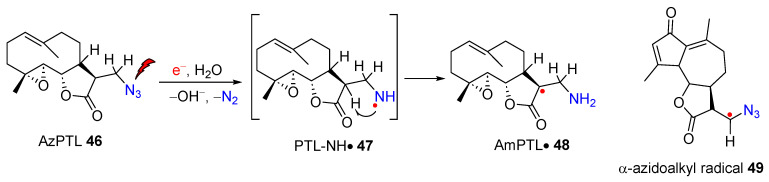
The intramolecular formation of the neutral tertiary carbon-centered radical **48** from azido-PTL **46**. Structure of α-azidoalkyl radical **49** from azido-DhL.

The general character of the site-specific generation of NCRs from azide group has been also illustrated with other natural products, such as sesquiterpene lactones [e.g., parthenolide (PTL) and dehydroleucodine (DhL)] [86]. The addition of radiation-produced electrons to azido-PTL **46** leads to the formation of highly reactive oxidizing aminyl radical **47**, which transforms into a stable α-carbonyl-stabilized tertiary C-centered radical AmPTL· **48** via [1,3]-hydrogen shift (Figure 7). Remarkably, the radiation of azido-DhL produces the corresponding aminyl radical, which, after bimolecular H-atom abstraction from substrate Azido-DhL, generates α-azidoalkyl radical **49**. Interestingly, no iminyl radicals have been detected upon the radiation of these sesquiterpene lactones. Azido-PTL and azido-DhL significantly suppressed proliferation rate and colony-forming ability in MCF-7 cells [86]. The azido-PTL in combination with radiation restricted its colony-forming ability to a greater extent than the PTL itself. The radiosensitization has been attributed to the increased reactive oxygen species (ROS) generated by the radicals produced from the azido group.

### 2.2. From Azido-Modified Nucleobases

The synthetic ability of the regioselective incorporation of the azido group at chemically distinctive positions of nucleobases (e.g., C5/C6 of pyrimidine bases or C2/C6 of pyrimidine moiety as well as C8 of imidazole moiety of purine bases [87]) provides a possibility for the selective generation of NCRs under the reductive conditions of dissociative electron attachment. It is noteworthy that often, it is challenging to selectively generate and elucidate the same specific NCR species during radiation and/or other oxidative conditions used during ionization events of non-azido nucleobases.

**Figure 8:**
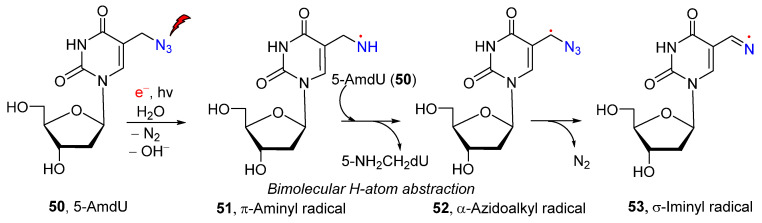
The formation of the neutral aminyl radical **51** by radiation-produced prehydrated electron attachment to 5-AmdU and its subsequent H-atom abstraction reaction with 5-AmdU to form iminyl radical **53** via the intermediary α-azidoalkyl radical **52** [88].

Wen et al. have employed 5-azidomethyl pyrimidine derivatives, such as AmdU **50** and AmdC, to study the radiation-mediated formation of RNH· and its subsequent reactions for potential anticancer properties (Figure 8) [88]. The authors hypothesized that the incorporation of azido-modified nucleosides into genomic DNA would augment radiation-induced damage in cells owing to the reactions of aminyl radicals under hypoxic conditions and therefore act as potential radiosensitizers. Their findings revealed that radiation-produced electron addition to 5-AmdU **50** generates the π-aminyl RNH· **51**. Radical **51** then undergoes an intermolecular reaction with 5-AmdU, abstracting the H-atom from the CH_2_N_3_ group at the C5 of 5-AmdU, to form the α-azido carbon radical **52**. The subsequent loss of N_2_ from **52** generates the thermodynamically more stable σ-iminyl radical [15,89] (R=N·) **53**. The AmdU and AmdC phosphates incorporate into DNA fragments in polymerase-catalyzed reactions, and AmdU demonstrated effective radiosensitization in EMT6 tumor cells in the presence or absence of oxygen [88]. AmdU has been also metabolically incorporated into DNA in living cells for the click labeling of DNA [90], and its 5′-triphosphate was found to be the substrate for DNA polymerases and PCR amplification [91]. Moreover, its 5′-triphosphate prodrug was shown to enhance its incorporation into the DNA of wild-type cells and animals [22]. Thus, AmdU can serve a dual purpose of labeling tumor cells prior to, during, or after radiotherapy, and it may radiosensitize the tumor during radiotherapy.

**Figure 9:**
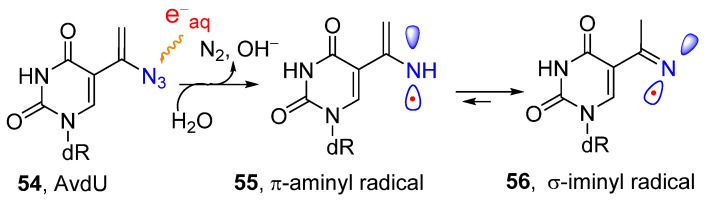
Tautomerization of π aminyl radical, generated from AvdU, to σ-iminyl radical [88].

The addition of a radiation-produced electron to 5-(1-azidovinyl)-2′-deoxyuridine (AvdU, **54**) also generates π-RNH· **55**, which undergoes facile tautomerization to thermodynamically more stable σ-iminyl radical **56** (Figure 9) [88]. One-electron attachment to the cytidine analog (AvdC) proved that the formation of the aminyl radical and its tautomerization to the iminyl radical has a general character and occurs independently of the nucleobase [88]. Owing to the high concentrations of various free radical scavengers in cells [92], the bimolecular conversion of π-RNH· to σ-R=N· from 5-azidomethyl nucleosides (e.g., AmdU) is unlikely to take place. However, the tautomerization of the π-RNH· to σ-R=N· from 5-azidovinyl nucleosides (e.g., AvdU) should occur even in cells. Therefore, it was expected that the π-RNH· from AmdU could augment radiation damage more effectively than the σ-R=N· from AvdU. The experiments in EMT6 tumor cells indeed showed the higher radiosensitizing effect of AmdU compared with that of AvdU [88].

**Figure 10:**
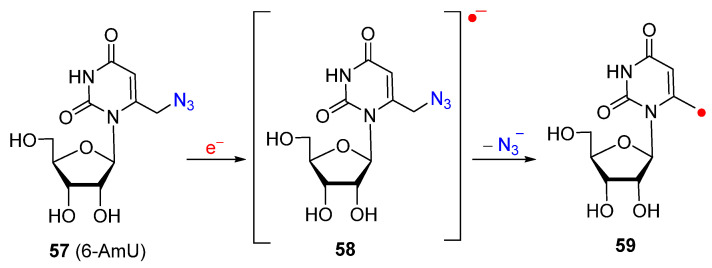
The formation of allylic radical **59** via the unexpected loss of azide as an anion (N_3_¯) from the azide anion radical intermediate **58** via dissociative electron attachment [76].

Distinct pathways of dissociative electron attachment have been observed in 6-azidomethyl uracil nucleosides [93] compared to their 5-azidomethyl counterparts (Figure 10) [76]. Contrary to the results with 5-AmdU [88], where radiation-mediated prehydrated electrons in the absence of oxygen led to π-aminyl and σ-iminyl radicals (see Figure 8), radiation-produced electron addition to 6-azidomethyluridine (6-AmU, **57**) leads to the unexpected loss of azide as an anion via dissociative electron attachment from the initially formed azide anion radical intermediate (**58**, U-6-CH_2_-N_3_·¯) or transient radical anion to generate the C6 allylic radical **59** [76].

**Figure 11:**
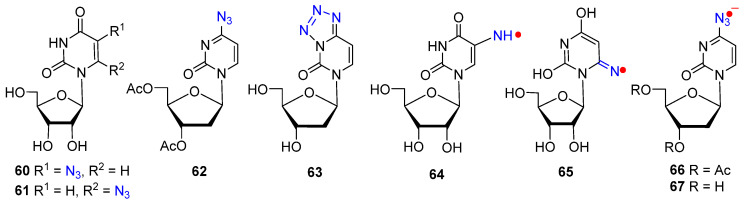
Nitrogen-centered radicals formed via dissociative electron attachment to 5-azidouridine **60**, 6-azidouridine **61**, and 4-azidopyrimidine analogue **62** and its cyclic tetrazolo derivative **63**.

The characterization of nitrogen-centered radicals formed via dissociative electron attachment to the azido group directly attached to a nucleobase, such as in 5-azidouridine **60**, 6-azidouridine **61**, and 4-azidopyrimidine analogue **62**, which have demonstrated how the presence of the azido group at different positions in the pyrimidine base (*meta* to N1 and N3 in **60** and *ortho*/*para* to N1 and N3 in **61** and **62**) distinctly affects the nature and stability of the nitrogen-centered radicals (Figure 11) [94]. The formation of (*i*) RNH· (**64**) from **60**, (*ii)* R=N· (**65**) from **61**, and (*iii*) RN_3_· ^−^ (**66**) from **62**, after gamma-irradiation at 77 K, was observed. Moreover, tetrazolocytidine **63** (a cylic derivative of **62**), upon irradiation, also produced azide anion radical **67** [94,95].

**Figure 12:**
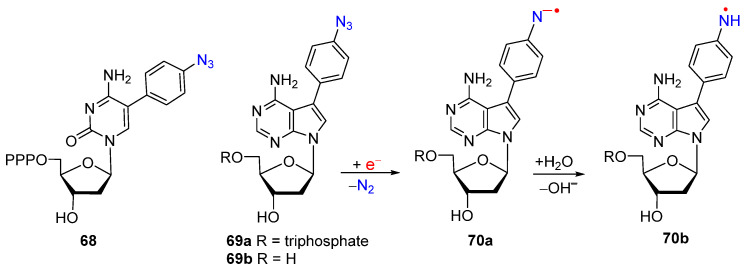
Azidophenyl-modified nucleosides and their electrochemical reduction on mercury surface to the aminyl radicals.

Hocek’s group developed azidophenyl (AzP)-labeled 5-(4-azidophenyl)-2′-deoxycytidine **68** and 7-(4-azidophenyl)-7-deaza-2′-deoxyadenosine **69a** triphosphates (dAAzPTP and dCAzPTP) as substrates for the enzymatic labeling of double- or single-stranded DNA (Figure 12) [96,97,98]. These novel electroactive azidophenyl-modified nucleotides and DNA gave strong signals in voltammetric studies at −0.9 V due to the reduction of the azido group, outside the potential region around −1.5 V, where natural bases are reduced. The proposed mechanism for the electrochemical reduction of dAAzP **69b** on mercury surface involves the one-electron reduction of the azido-group to nitrene anion radical **70a** accompanied by dinitrogen release. The nitrenium ion-radical is then protonated to form the π-aminyl radical **70b**, which is stabilized by an aromatic phenylene linker bound to nucleobase. The subsequent electrochemical reduction of the aminyl radical by one electron and one proton yield the amine [98]. The nucleosides with the new AzP redox label are not only suitable for the electrochemical detection but can also be transformed to another redox label or silenced through the click reactions.

## 3. Nitrogen-Centered Radicals Generated on Non-Azido Nucleobases

**Figure 13:**
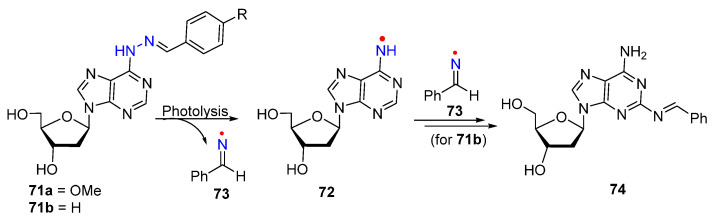
Plausible generation of dAdo-6-*N*-aminyl radical **72** by photolysis of dAdo-6-*N*-arylhydrazones **71a** and **71b** and its subsequent chemistry to form 2-imino-dAdo derivative **74** [99].

Approaches for the generation of the similar NCRs from the selectively modified adenine, guanine, and cytosine substrates “armed with radical initiators” other than the azido group have also been developed [99,100,101]. Thus, Wagner’s group demonstrated the formation of N^6^-aminyl radical **72** with the photolysis of 6-*N*-arylhydrazones of 2′-deoxyadenosine **71a** and **71b** in the presence of H-donors, such as glutathione (GSH; Figure 13) [99]. Specifically, 6-*N*-(4-methoxyphenyl)hydrazone **71a** was more efficient for NCR formation compared to phenylhydrazone **71b**. Upon the photolysis of the latter, N^6^-aminyl radical **72** and benzylidene iminyl radical **73** were postulated to be generated via homolytic cleavage as the subsequent H-atom abstraction by **72** and **73** from GSH-produced 2′-deoxyadenosine (dAdo) and benzaldehyde. The decomposition of N^6^-aminyl radical **72** in the excess of dAdo revealed the generation of 6-amino-2-imino product **74** (45%) via the recombination of **72** and **73** at the C2. These studies reveal the potential new path for the selective formation of adenosyl-6-*N*-aminyl radical **72** under neutral conditions.

**Figure 14:**
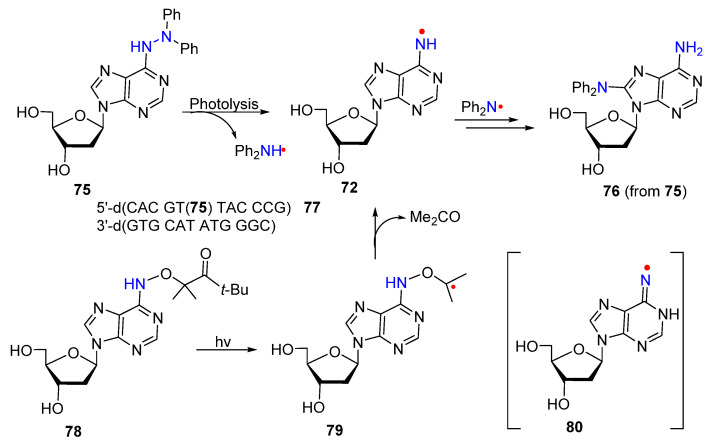
The plausible generation of dAdo-6-*N*-aminyl radical **72** by the photolysis of ketone **78** as well as hydrazine **75** and the subsequent chemistry to form 8-amino-dAdo derivative **76**.

Greenberg’s group identified the aminyl radicals with the photolysis of the hydrazine modifications at the 6-*N*-positon of 2′-deoxyadenosine and 2-*N*-positon of 2′-deoxyguanosine and showed the compatibility of these hydrazine analogues for solid-phase oligonucleotide synthesis to study DNA–hole transfer processes [100]. Specifically, the photolysis of hydrazine dAdo precursor **75** produced C8-diphenylamino-substituted dAdo **76** in addition to dAdo (Figure 14). The identification of these photolyzed products indicated that N^6^-aminyl radical **72** is initially generated from **75** with the loss of the diphenyl aminyl radical (Ph_2_N·). The subsequent reaction between **72** and Ph_2_N· produced **76**. The compatibility of these hydrazines for solid-phase oligonucleotide synthesis was proven via the generation of dodecameric duplex **77**, as T_m_ decreased significantly compared to its dAdo duplicate [100]. Although spectroscopic evidence (UV, MS) and (in)direct synthetic proofs were presented to indicate the formation of the aminyl radical **72**, there was no conclusive EPR results unequivocally characterizing this radical due to a very poor rate of fragmentation in glassy systems at low temperature. It is noteworthy to point out that in Wagner’s studies of the in situ-generated benzylidene iminyl radical, **73** was postulated to add to the C2 position of the purine ring (π-deficient pyrimidine ring) to give **74**, whereas in Greenberg’s investigations, the diphenyl aminyl radical adds to the C8 position (π-excessive imidazole ring) to produce **76**.

The photocleavage of ketone **78** and the β-fragmentation of the initially formed alkyl radical **79** also led to aminyl radical **72** (Figure 14) [101]. The formation of **72** was followed by laser flash photolysis (LFP), which yielded a transient with λ_max_ ≈ 340 nm and a broader weaker absorption centered at ~560 nm. The calculations indicate that the iminyl tautomer **80** of **72** is 13.0 kcal/mol higher in energy in the gas phase and its forms must rapidly isomerize to **72**. Precursor **78** has been incorporated into DNA fragments, and the site-selective generation of the neutral purine nitrogen radical **72** was shown to produce tandem lesions. The involvement of 2′-deoxyadenosin-6-*N*-yl radical **72** in this process could be detected because it was independently generated from the synthetic precursors [102,103,104].

**Figure 15:**
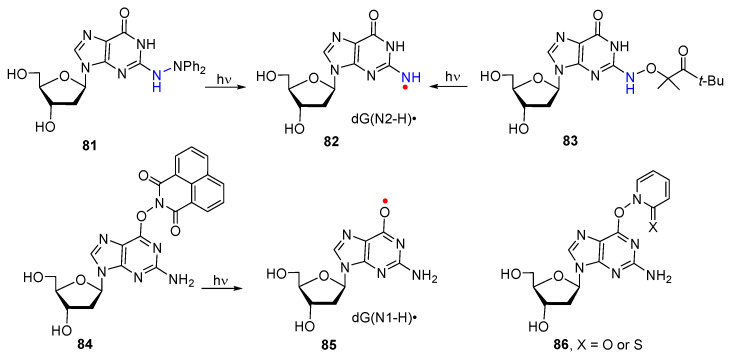
Photochemical generation of guanosyl radicals from synthetic precursors.

The photolysis of dGuo hydrazine **81** provided strong evidence for the formation of dGuo-N^2^-yl radical **82** as determined by the generation of 2′-deoxyguanosine (Figure 15) [100]. The independent generation and time-resolved detection of radical **82** was developed by the photolysis of ketone radical initiator **83** [81]. The LFP experiments showed no evidence for the water-assisted tautomerization of dG(N2-H)· **82** to dG(N1-H)· **85** within hundreds of microseconds, supporting the theoretical prediction by von Sonntag [92,105]. This observation suggests that the generation of dG(N1-H)· via dG(N2-H)· following hydrogen atom abstraction from dG and subsequent tautomerization is unlikely [81]. The formation of the N1-yl radical G(N1-H)· **85** has been indirectly observed via the one-electron oxidation of N-(aryloxy)naphthalimide **84** [106]. Photolabile N-hydroxypyrid-2(1*H*)-one or thione **86** were also used for guanine radical generation [107,108]. It is noteworthy that preliminary results on the addition of a radiation-produced electron to 2-azido-2′-deoxyinosine produces the complex EPR signals, and subsequent UV photoexcitation leads to the mixture of radicals, including the possible formation of sugar radicals rather than the elusive guanyl aminyl radical **82** [95].

**Figure 16:**
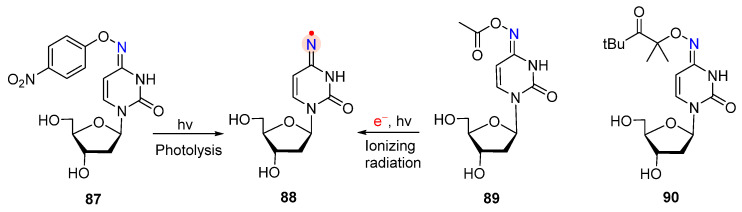
The generation of 2′-deoxycytidin-N4-yl iminyl radical **88** by photolysis and radiation-produced prehydrated electron attachment approaches.

The Greenberg’s group reported the photochemical generation of σ-iminyl radical 2′-deoxycytidin-4-*N*-yl **88** from a nitrophenyl oxime precursor **87** and the synthesis of oligonucleotides containing **87** for DNA incorporation (Figure 16) [109]. It is worth noting that the attempted photolytic generation of **88** from oxime esters **89** and **90** was unsuccessful due to instability and poor fragmentation. The formation of the iminyl radical **88** from **87** was confirmed (in)directly via LFP and transient UV-absorption spectroscopy [109]. Photolysis studies also revealed **88** can recombine with the aryloxyl radical to regenerate **87**, undergo C5-C6 addition to produce diradicals, generate deoxycytidine via reduction, or react under aerobic conditions to generate other radicals/products. Iminyl radical **88** was also independently generated (and characterized by EPR) via single-electron transfer to oxime ester **89** [110]. Like with adeninyl radical **72**, tandem lesion formation from cytosinyl radical **88** is traceless because it is reduced to dC during the process. However, unlike **72**, an isolated **88**, which is a stronger oxidant, directly oxidizes dG. In this regard, **88** is more similar to a nucleoside alkyl aminyl radical, such as the one generated from AZT [79,109].

**Figure 17:**
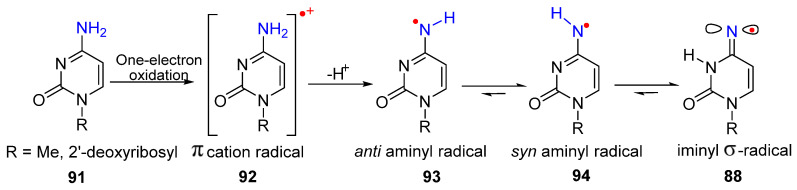
Formation of cytidine π-aminyl radicals **93** and **94** that tautomerize to σ-iminyl radical **88** upon one-electron oxidation of cytosine nucleobase [111].

One-electron oxidation and ESR studies of 1-methylcytosine and 2′-deoxycytidine **91** showed that the cytidine cation radical **92** preferentially deprotonates to form an aminyl radical **94** *syn* to the carbonyl moiety (Figure 17). The tautomerization of *syn*-aminyl radical also leads to the iminyl σ-radical **88** [111,112]. Interestingly, contrary to these findings, [109,110,111] the one-electron reduction (conditions for DEA) of azide precursors at the C4 positions of pyrimidine bases (**62** or **63**) led to the formation of anion radicals **66** or **67** (see Figure 11) [94,95].

## 4. Conclusions

Nucleosides with a regioselectively inserted azido group at the sugar or base moiety are unique precursors for the generation of nitrogen-centered radicals (NCRs) under enzymatic, electrochemical, and dissociative electron attachment (reductive) conditions. The azidonucleoside substrates under reductive conditions produce the same NCR species that are observed upon radiation and/or other oxidative conditions of parent nucleosides, making them unique and desired precursors. The generation of the NCRs from azidonucleosides and their subsequent reactivity depends on the nucleophilicity/electronegativity as well as redox potentials of nucleobases, which define the fragmentation of the initially formed transient negative ions [(RN_3_)· ^−^]* and their dissociation from the release of N_2_ (to form π-aminyl radicals, RNH·) or loss of N_3_^−^ (to form R·; Scheme 1). Due to their reactivity, the RNH· undergo a plethora of reactions, including selective hydrogen atom abstractions from the phosphate–sugar backbone or from the nucleobases as well as their addition to the double bond in the proximate base moiety.

The azidonucleosides have shown significant promise in their application as radiosensitizers for increasing the efficacy of tumor radiochemotherapy. For instance, 5-azidomethyl-2′-deoxyuridine demonstrated effective radiosensitization in EMT6 tumor cells in the presence or absence of oxygen. On the molecular level, it was found that the inactivation of RDPR by 2′-azido-2′-deoxynucleotides was accompanied by the appearance of new EPR signals for a nitrogen-centered radical resulting from the reaction of azide with a protein thiyl radical. Thus, this overview summarizes the investigations outlining the involvement and role of azidonucleoside-produced NCRs that are involved in the first signaling steps (i.e., radicals leading to stable damage products) that affect cellular functions.

Studies on the nucleoside substrates “armed with radical initiators” other than an azido group placed selectively on the exo-amino group of adenine, guanine, and cytosine have led to the generation/detection of similar NCRs under photolytic conditions. These non-azido analogues were found to be compatible for the solid-phase synthesis of DNA fragments, and photolysis was used to study DNA interaction, stability, and hole transfer.
